# Empowerment among adult patients with type 2 diabetes: age differentials in relation to person-centred primary care, community resources, social support and other life-contextual circumstances

**DOI:** 10.1186/s12889-021-10855-0

**Published:** 2021-05-01

**Authors:** Nina Simonsen, Anne M. Koponen, Sakari Suominen

**Affiliations:** 1 Folkhälsan Research Center, Public Health Research Program, P.O. Box 211, 00251 Helsinki, Finland; 2 Department of Public Health, University of Helsinki, Helsinki, Finland; 3 Department of Public Health, University of Turku, Turku University Hospital, Lemminkäisenkatu 1, Turun yliopisto, 20014 Turku, Finland; 4 School of Health and Education, University of Skövde, Skövde, Sweden

**Keywords:** Type 2 diabetes, T2D, Empowerment, Quality of care, Chronic care model, PACIC

## Abstract

**Background:**

Rising prevalence of type 2 diabetes (T2D), also among younger adults, constitutes a growing public health challenge. According to the person-centred Chronic Care Model, proactive care and self-management support in combination with community resources enhance quality of healthcare and health outcomes for patients with T2D. However, research is scarce concerning the importance of person-centred care and community resources for such outcomes as empowerment, and the relative impact of various patient support sources for empowerment is not known. Moreover, little is known about the association of age with these variables in this patient-group. This study, carried out among patients with T2D, examined in three age-groups (27–54, 55–64 and 65–75 years) whether person-centred care and diabetes-related social support, including community support and possibilities to influence community health issues, are associated with patient empowerment, when considering possible confounding factors, such as other quality of care indicators and psychosocial wellbeing. We also explored age differentials in empowerment and in the proposed correlates of empowerment.

**Method:**

Individuals from a register-based sample with T2D participated in a cross-sectional survey (participation 56%, *n* = 2866). Data were analysed by descriptive statistics and multivariate logistic regression analyses.

**Results:**

Respondents in the youngest age-group were more likely to have low empowerment scores, less continuity of care, and lower wellbeing than the other age-groups, and to perceive less social support, but a higher level of person-centred care than the oldest group. Community support, including possibilities to influence community health issues, was independently and consistently associated with high empowerment in all three age-groups, as was person-centred care in the two older age-groups. Community support was the social support variable with the strongest association with empowerment across age-groups. Moreover, vitality was positively and diabetes-related distress negatively associated with high empowerment in all age-groups, whereas continuity of care, i.e. having a family/regular nurse, was independently associated in the youngest age-group only.

**Conclusion:**

Person-centred care and community support, including possibilities to influence community health issues, supports empowerment among adults with T2D. Findings suggest that age is related to most correlates of empowerment, and that younger adults with T2D have specific healthcare needs.

**Supplementary Information:**

The online version contains supplementary material available at 10.1186/s12889-021-10855-0.

## Introduction

The rising prevalence of type 2 diabetes (T2D) has been described as a pandemic, and thus a growing public health challenge worldwide [1]. In 2017, about 425 million adults in the age range between 20 and 79 years had diabetes, and the number is expected to rise to 629 million adults by 2045 [2]. T2D is most common in older adults – and, for example in Finland, the prevalence of diabetes among older adults (≥ 65 years) nearly tripled in the years from 2000 to 2015 [3]. Nevertheless, it is increasingly common also among younger adults [1, 4] and studies, moreover, suggest that the disease course might be more severe and the level of psychosocial distress higher in this age group [5–7]. The main targets in the management of T2D are a good quality of life, mental well-being and achieving and maintaining recommended levels of metabolic control, in order to reduce complications. The main means to reach metabolic control rely on healthy lifestyle choices and disease self-management, including medication, if needed, and effective strategies for coping with stress [8]. People with T2D have to manage their own health in daily life and studies show that their own perception of their competence or self-efficacy to do this is associated with better self-management and outcomes of care [9–11]. Accordingly, there is a need for reorienting primary healthcare towards self-management support with a focus on self-efficacy or patient empowerment [12, 13]. Primary healthcare faces great challenges in meeting the needs of the growing number of people with T2D. Moreover, higher prevalence among younger adults implies that patient age increasingly needs to be considered when tailoring healthcare services and support programs [5, 7]. However, there is still limited knowledge about the influence of age on diabetes-related variables.

New models of service delivery, different from the reactive acute-oriented care, have been advocated and initiated in primary healthcare to improve quality of chronic illness care. One highly recognized and recommended model to improve quality of diabetes care is the Chronic Care Model (CCM) [14–16]. CCM strongly emphasizes person-centredness and self-management support – including collaborative goal setting, problem solving and follow-up. According to the model, an informed, empowered patient and a prepared, proactive multidisciplinary care team are vital elements of high-quality care [14, 17]. Overall, the evidence concerning the potential of the CCM to improve outcomes of care is growing [15, 18–20]. However, far less is known about process outcomes of CCM-concordant care, such as self-efficacy for disease management and perceived social support – knowledge, which would be needed for further improvements in care processes [15].

Patient empowerment has in recent years been proposed as an important quality indicator in its own right in evaluating both healthcare processes and outcomes as concerns chronic conditions such as T2D [13, 21]. Patient empowerment definitions mostly focus on the (inherent) capacity of patients to be responsible for their own health, to make decisions about their health and behaviour, and to gain (greater) control over life-circumstances that relate to health [13, 22, 23]. Empowerment is one of the key principles in health promotion according to WHO [24] and in that context it is emphasized that people should be able to bring about changes, not only as relates to their personal behaviour, but also with regard to their social environments and the organizations that affect their lives [25, 26].

Today, there is some evidence that perceived quality of chronic illness care (CCM-concordant person-centred care) and perceived social support are independently and positively associated with empowerment among patients with T2D [21]. However, the relative importance of different support sources, and, most notably, community support, to patient empowerment is not known. The CCM proposes that community resources, in addition to healthcare personnel’s self-management support, may enhance care [14, 20, 27, 28]. Still, few studies have addressed the community resource component of CCM [15]. Moreover, there is a lack of studies that include diabetes patients’ possibilities to influence health and life quality issues in the community, i.e. the opportunity to make a positive impact in the community – an important aspect of the process of empowerment according to the WHO definition [25, 26].

Given the limited knowledge about the association of age with diabetes patients’ perceptions of quality of care, with patient-reported outcomes such as empowerment, and with other important diabetes-related life-contextual factors, we set out to explore this in a Finnish context. Moreover, to our knowledge, earlier studies have not examined whether there are age-related differences with respect to correlates of empowerment among patients with T2D. Besides person-centred care and various social support sources we included other quality of care (continuity of care, diabetes counselling), psychosocial and background variables, which may be associated with patient empowerment [21].

Thus, the aim of this study, in a Finnish register-based sample of patients with T2D, was to develop further the understanding of chronic illness care and empowerment in diabetes care by:

Firstly, in three age-groups (27–54, 55–64 and 65–75 years) exploring age differentials in patient empowerment, perceptions of quality of care, diabetes-related social support and psychosocial wellbeing; and, secondly, examining in the three age-groups whether person-centred care and diabetes-related social support, including community support and possibilities to influence community health issues, are associated with patient empowerment, when controlling for background and possible confounding factors (i.e. other quality of care indicators, psychosocial wellbeing).

## Methods

### Design and setting/ data collection

Data was collected through a mail survey in 2011. The study is part of a larger study of quality of care in T2D in five municipalities in Southern and Central Finland (the ‘Good Diabetes Care’ -Study). The sample was drawn from a nationwide register of all persons with entitlement to a special reimbursement for medicines used in the treatment of T2D, kept by the Social Insurance Institution of Finland (SII). SII is a government agency in charge of settling benefits under national social security programs.

In all, 7575 persons fulfilled the inclusion criteria, including having entitlement to a special reimbursement for medicines used in the treatment of T2D, born in 1936–1991 (20–75 years), and Finnish as native language, as well as one of the five study municipalities as place of residence [7]. Among these, a sample of 5167 persons was collected from the register based on a power-analysis, i.e., all persons with T2D from the 3 small municipalities (in order to make it possible to carry out comparisons between municipalities for other research questions in the Good Diabetes Care -Study) and 2000 randomly from each of the two larger municipalities. In this sample, there were 2962 (57%) men and 2205 women (43%), which corresponded to sex rates in the total population of patients with T2D in the five study municipalities.

The questionnaire was mailed to respondents in September 2011 by the SII with a reply-paid envelope addressed directly to the research institute. In October, a reminder to non-respondents was sent out, and another reminder with a new copy of the questionnaire in November. The Ethical Committee of the Hjelt Institute, University of Helsinki, and the SII approved the study.

### Measures

#### Outcome variable

We used the short form of the *Diabetes-Empowerment Scale* (DES-SF) as the outcome variable. It is an 8-item validated measure that assesses overall diabetes-related psychosocial self-efficacy, including assessing need for change, developing a plan, overcoming barriers, asking for support, supporting oneself, coping with emotion, motivating oneself and making diabetes care choices appropriate for one’s priorities and circumstances [29, 30]. The items take the form “In general I believe that I…” and are answered on a 5-point scale ranging from ‘strongly disagree’ to ‘strongly agree’, with a Cronbach’s alpha reliability of 0.86 in our data. We dichotomized the scale by the median.

#### Main independent variables

We measured person-centred care with the Patient Assessment of Chronic Illness Care, i.e., *PACIC* scale – a quality of chronic illness care indicator – which has been developed to evaluate CCM-concordant care from patients’ perspective [31] and advocated as a patient-reported measure of person-centred coordinated care [32]. It is a 20-item validated measure, comprising five subscales: patient activation, decision support, goal setting, problem solving and coordination/follow-up. An example item is: “I was asked for my ideas when we made a treatment plan”. Earlier studies have proposed the use of the PACIC total score as an overall experience of chronic illness care [33–35]. The Finnish validated version of the PACIC scale [36] uses an extended 12-months’ time frame, giving patients a possibility to base their responses on a longer period of care. Each item on the measure is rated on a five-point scale (from ‘almost never’ to ‘almost always’). The overall PACIC score is an average across all 20 items. Higher scores indicate higher quality of care. The Cronbach’s alpha in our sample was 0.94 for the total scale.

To measure *Diabetes-related social support and possibilities to influence community health issues* we used the 12-item measure by Toljamo [37] assessing diabetes-related help and support received from relatives, friends and health care personnel on a 5-point scale ranging from ‘strongly disagree to ‘strongly agree’. An example item is: “My family and friends take care of things for me when necessary”. The Diabetes Social Support scale of Toljamo is based on scales developed by, for example, Brandt and Weinert [38]. In addition, we included three items (see Additional file 1): two items measuring support received from and in the community regarding diabetes self-care, adapted from the community resources component of the Resources and Support for Self-management (RSSM) instrument [39], and one item assessing possibilities to influence factors related to health and life quality in one’s community, constructed based on the WHO definition of empowerment where people’s greater control over actions and decisions that affect their health is in focus [25]. Based on a principal component analysis of all the 15 items, we found three factors: social support from one’s family and friends i.e. *family and friend support* (4 items; alpha 0.78), social support from peers*,* i.e. *peer support* (2 items; alpha 0.82) and social support from the community and possibilities to influence community health issues, i.e. *community support and possibilities to influence* (6 items; alpha 0.83). The community support and possibilities to influence factor included two items on informational support from healthcare personnel, two items on support from the community, i.e. one item on support in terms of economic support and one in terms of overall support, one item on sufficiency of activities in the community that support one’s self-management, and one item on possibilities to influence factors related to health and life quality in one’s community. One of the items on the Toljamo scale (related to follow-up visits in the diabetes clinic being very important for getting information) did not load on any factor, and two of the items measuring negative aspects of social support, which loaded on one factor, had a low Cronbach’s alpha (i.e. 0.39) and we did not include them in the analyses. Higher scores indicate higher perceived support.

### Additional independent variables measuring quality of care and psychosocial wellbeing

*Diabetes counselling* was measured with questions concerning the sufficiency of information, advice and guidance related to different aspects of diabetes self-management gotten in one’s principal primary healthcare centre, for example: 1) diabetes as a disease, 2) diabetes medication …..15) suitable physical activity (15 items; range 1 = not at all…3 = sufficiently; see Additional file 1).

The indicators for *continuity of care*, as concerned diabetes care, included having a family/regular doctor and/or a family/regular nurse (the questions answered with 1 = no/don’t know, 2 = yes).

We measured *emotional well-being* and *energy/vitality* with scales from the Finnish validated version of the RAND-36-Item Health Survey, 1.0 (range 0–100) [40, 41] that measure experiences during the last 4 weeks with 5- respectively 4-item scales. The items take the form: “How much of the time during the past 4 weeks…”. An example item is: “How much of the time during the past 4 weeks did you have a lot of energy?”. Cronbach’s alphas were 0.84 respectively 0.85 in our data. Higher scores indicate better emotional well-being, respectively higher felt energy or vitality.

*Life stress* was measured with a 10-item scale (see Additional file 1) measuring experienced stress during the last year in 10 life areas, for example, due to one’s health or economic situation (range 1 = none…4 = very much). Based on the Living with Diabetes Study [42].

*Diabetes-related distress* was measured with a 6-item scale (see Additional file 1) measuring how often self-management of diabetes (monitoring blood-sugar levels, measuring blood-pressure, checking one’s feet, taking one’s medicine, eating healthily and engaging in physical activity) felt burdensome or difficult (range 1 = almost never….4 = almost always).

Furthermore, we included the following *background items*: sex, age, marital status, professional education, diabetes medication, years since diagnosis, and health care provider. The focus was on three age groups, that is, older (65–75 years), ‘middle aged’ (55–64 years) and younger (27–54 years) respondents. The age-limit for the oldest age group was based on the common age for retirement in Finland (i.e. 65 years). For the youngest age group, the higher age-limit was at the outset based on early onset of T2D (defined as ≤45 years) and the lower age-limit was 20 years. However, due to the relative low number of persons of this age in our sample, we raised the age-limit to 54 years. Also, the youngest to answer were 27 years old. Thus, we defined the youngest age group as respondents who were 27–54 years.

All continuous scales are averaged sum-scales (i.e. DES-SF, PACIC, Diabetes-related social support, diabetes counselling, emotional well-being, energy/vitality, life stress and diabetes-related distress). Respondents were included in the analyses if they had answered to at least 85% of the PACIC scale items (i.e. 17 of 20 items) to ensure inclusion of the different dimensions of the scale, as well as 70% of the other scale items. Higher values correspond to the meaning of the scale. All data are self-reported.

### Statistical analyses

In descriptive analyses, to test for age-related differences in background, dependent, main independent and additional independent variables, Chi-square-test, one-way analysis of variance (ANOVA) and Kruskal-Wallis test were applied, as appropriate. Thereafter, baseline associations between background, independent variables and dependent variable were tested with Chi-square-test and *t*-tests. In the final analyses, first, univariate and then multivariate logistic regression analyses were performed separately for the three age groups. We also tested the interaction terms between main independent variables and age groups on diabetes empowerment in adjusted regression models.

Before regression analyses, correlations between variables were explored with Spearman’s correlation coefficients. Variables to be included in the multivariate regression models were selected based on their significance in the baseline analysis of associations between them and diabetes empowerment, as well as based on correlations between independent variables to avoid multicollinearity. In the final multivariate logistic regression analyses, we tested whether person-centred care (PACIC), continuity of care and diabetes-related social support were independently associated with diabetes empowerment, when controlling for important psychosocial (diabetes-related distress, energy/vitality) and background (sex, marital status, education) variables. We included the variables stepwise in the regression models. Listwise deletion was used for missing values. All statistical analyses were performed using SPSS version 25. The significance level was set at *p* < 0.05.

## Results

### Background characteristics of the sample

Responses were received from 2866 respondents (56% response rate). The response rate was highest in the oldest age group (63%), lower in the middle age group (55%) and lowest in the youngest age group (36%). The mean age of respondents in the study sample was 63 years (SD 8, range 27–75 years), 56% were male, and 41% had a higher professional education, meaning college, polytechnic or university education. The mean duration of diabetes was 8 years (SD 6), and the majority (75%) used oral diabetes medication only. Moreover, for the majority (77%) municipal primary healthcare centres were the main provider of diabetes care. Of respondents, 84% had been for over 2 years, and 95% at least for 1 year, in care at their current primary care centre.

In the sample, 13% were 27–54 years, 38.7% were 55–64, and 48.3% 65–75 years. In the youngest age group (27–54 years), 70% had been diagnosed with T2D at the age ≤ 45 years. In the other age groups, as well, there were persons with early onset diabetes: 11% of the middle age group, and 4% of the oldest age group.

### Age differentials in study variables: descriptive findings

Table 1 provides background data on the study sample across age groups. There were differences between the age groups on all background factors except sex. The youngest age group, as compared with the two older age groups, were more often single and more often had a higher professional education; they more seldom had had diabetes over 10 years, more seldom used oral diabetes medication only, and more seldom had a municipal healthcare centre as the primary care organization responsible for their diabetes care.

**Table 1:** Background factors of respondents in the study sample, across age groups (%)

Characteristic	Age 27–54 ***n*** = 341	Age 55–64 ***n*** = 1019	Age ***≥*** 65 ***n*** = 1270	***P***-value
	%	%	%	
Sex				
Men	58.4	56.9	54.7	ns.
Women	41.6	43.1	45.3	
Professional education				
Upper secondary education (vocational school) or less	50.7	57.2	62.2	***
Higher education (college, polytechnic, university)	49.3	42.8	37.8	
Marital status				
Single	21.2	11.5	4.8	***
Married/cohabiting	60.2	68.0	68.3	
Widowed/divorced	18.6	20.5	26.8	
Duration of diabetes				
1–3 years	32.2	20.3	15.7	***
4–10 years	50.8	57.0	50.5	
More than 10 years	17.0	22.7	33.9	
Medication				
Oral drugs only	64.1	73.8	77.8	***
Oral drugs + insulin/insulin only	33.1	24.7	21.6	
Other (e.g. GLP-1 analog)	2.8	1.4	0.7	
Service provider responsible for care of diabetes:				
Municipal healthcare centre	58.9	65.2	91.4	***
Occupational healthcare service	38.9	29.5	4.4	
Private healthcare centre	2.2	5.3	4.2	

*Chi*^*2*^*-test*

*P*-value for statistical significance between age groups: ***p ≤ .001, ns. non-significant

As shown in Table 2 there were also significant differences between the age groups on the dependent variable, and on most independent variables. The respondents in the youngest age group (27–54 years), as compared with the two older ones, more often had a low empowerment level (*p* < 0.01/ *p* = 0.001, respectively) and a higher life stress and diabetes-related distress level (*p* < 0.001); they had less energy and lower emotional well-being (p < 0.001). Those in the youngest age group, as compared with the oldest one, perceived that they had less family and friend support (*p* < 0.01), and less community support and possibilities to influence (*p* < 0.01); however, they had higher PACIC scores (p < 0.001). The youngest age group more seldom had a family/regular physician (*p* < 0.05) as compared with the other age groups, and more seldom a family/regular nurse as compared with the oldest group (p < 0.05).

**Table 2:** Descriptive statistics of the scales included in the study, across age groups: % or mean (SD)

	Age 27–54	Age 55–64	Age ***≥*** 65	***P***-value
**Scale**				
Empowerment (%)				
High (≥ 4)	48.4	57.7	58.4	**
Low (< 4)	51.6	42.3	41.6	
Continuity of care (%):				
-Family/regular physician				
No/don’t know	31.5	25.0	24.9	*
Yes	68.5	75.0	75.1	
-Family/regular nurse				
No/don’t know	53.5	50.1	46.2	*
Yes	46.5	49.9	53.8	
PACIC (range 1–5)	2.5 (0.9)	2.4 (0.9)	2.2 (0.8)	***
Social support (1–5)				
Community	3.3 (0.9)	3.4 (0.8)	3.5 (0.8)	**
Family and friends	3.7 (1.1)	3.8 (1.0)	3.9 (0.9)	*
Peers	3.1 (1.3)	3.2 (1.3)	3.3 (1.2)	ns.
Stress (1–5)	2.0 (0.5)	1.7 (0.5)	1.5 (0.4)	***
Diabetes-related distress (1–4)	2.0 (0.6)	1.8 (0.6)	1.6 (0.5)	***
Energy/vitality (0–100)	52.6 (23.9)	59.6 (22.8)	62.5 (21.5)	***
Emotional well-being (0–100)	65.2 (20.8)	71.8 (19.5)	74.8 (18.3)	***
Diabetes counselling (1–3)	2.5 (0.5)	2.5 (0.5)	2.5 (0.5)	ns.

*Chi*^*2*^*-test* for categorical variables and *One-way ANOVA or Kruskal-Wallis test* for continuous variables

*P*-value for statistical significance between age groups: *** *p* ≤ .001; ** *p* < .01; * *p* < .05; ns. non-significant

### Baseline associations between empowerment and background and independent variables

We performed preliminary analysis of baseline associations (Chi-square-test and *t*-tests) between study variables in the whole sample, in order to choose which variables to include in the regression analyses.

Sex (p < 0.01), age (p < 0.01), marital status (p < 0.05) and professional education (p < 0.01) were all significantly associated with empowerment, whereas duration of diabetes, diabetes medication and service provider responsible for diabetes care were not. Thus, sex, age, marital status and professional education were included in the regression analyses. Respondents with a high sense of empowerment, as compared with those with a low sense of empowerment, were more often female, older, married or cohabiting, and had a higher professional education level.

Furthermore, all psychosocial and quality of care indicators (i.e. proposed independent variables) were associated with diabetes empowerment. Respondents with a low sense of empowerment, as compared with those with a high sense of empowerment, had higher life stress and diabetes-related distress levels (*p* < 0.001), lower energy levels, and poorer emotional well-being (p < 0.001); they also perceived that they received less support from all three social support sources (p < 0.001). Moreover, they had lower PACIC scores (p < 0.001), were less satisfied with the diabetes counselling they had received in their primary healthcare centre (p < 0.001), and they more seldom had a family/regular physician and/or nurse (p < 0.001) and thus less continuity of care.

The bivariate correlations among the proposed independent variables showed high correlations between energy/vitality and emotional well-being (.77), between PACIC and diabetes counselling (.62), and quite high between life stress and energy/vitality (.49; all *p*-values < .001). Because of the high correlations, we only included energy/vitality in the regression analyses as a measure of mental well-being, and PACIC and continuity of care, but not counselling, as measures of quality of chronic illness care.

Of the variables we included for further analyses, PACIC correlated moderately with community support and possibilities to influence (.46; *p* < .001), and positively also with empowerment (0.22), peer support (.22), having a family/regular nurse (0.20) or doctor (0.19), and family and friend support (.17; *p* < 0.001 for all values) (Table 3). As regards correlations with empowerment: of all variables, empowerment correlated most strongly with community support and possibilities to influence (0.32), energy/vitality (0.30), family and friend support (0.26), PACIC (0.22) and peer support (0.21; p < 0.001 for all values).

**Table 3:** Bivariate correlations between study variables

	1	2	3	4	5	6	7	8	9	10	11
1.Sex (1 = man, 2 = woman)											
2. Marital status (1 = single, 2 = married/cohabiting)	−.17***										
3.Education (1 = lower education 2 = higher education)	−.02	.07***									
4.PACIC	−.07***	.07***	.01								
5.Family doctor (1 = no, 2 = yes)	.00	.04	−.09***	.19***							
6.Family nurse (1 = no, 2 = yes)	.05*	−.02	−.10***	.20***	.18***						
7.Community support	−.07***	.10***	−.03	.46***	.22***	.19***					
8.Family and friend support	−.00	.26***	−.02	.17***	.07***	.05*	.39***				
9. Peer support	.06**	.03	−.12***	.22***	.16***	.11***	.41***	.29***			
10.Energy/vitality	−.08***	.12***	.00	.15***	.07***	.05*	.31***	.31***	.19***		
11.Diabetes-related distress	.06**	−.11***	.02	−.07***	−.05*	−.03	−.22***	−.20***	−.14***	−.39***	
12. Empowerment (1 = low, 2 = high)	.05**	.05**	.05**	.22***	.10***	.09***	.32***	.26***	.21***	.30***	−.27***

***p ≤ .001; **p < .01; *p < .05

### Regression analyses of correlates of diabetes empowerment

As all background and independent variables were strongly associated with age, the regression analyses were done separately for the three age groups. In the univariate regression analyses, sex was associated with empowerment only among the oldest age group, and professional education only among the middle age group. Marital status was associated with empowerment among both the youngest and the middle age group. All other included variables were associated with empowerment, except having a family/regular nurse among the oldest age group.

In the multivariate regression models, in the youngest age group (27–54 years, Table 4), PACIC was significantly associated with diabetes empowerment when controlling for background characteristics and for continuity of care indicators. When the social support variables were included in the model, the significant association between PACIC and empowerment disappeared. In the final model, having a family/regular nurse, higher levels of community support and possibilities to influence, and higher energy levels were positively and diabetes-related distress negatively associated with empowerment.

**Table 4:** Multivariate logistic regression models on determinants of empowerment among patients with T2DM, age group: 27–54 years

	Model 1 OR (95% CI)	Model 2 OR (95% CI)	Model 3 OR (95% CI)	Model 4 OR (95% CI)	Model 5 OR (95% CI)
Sex:					
1. men (Ref.)	1.19 ns. (.73–1.93)	1.19 ns. (.72–1.96)	1.14 ns (.69–1.88)	1.08 ns (.64–1.83)	1.31 ns (.75–2.27)
2. women					
Marital status					
1.single/widowed/divorced (Ref.)	1.68* (1.04–2.74)	1.58 ns. (.96–2.6)	1.62 ns (.98–2.69)	1.28 ns (.72–2.28)	1.24 ns (.69–2.25)
2.married/cohabiting					
Professional education					
1.lower education (Ref.)	1.44 ns. (.90–2.32)	1.43 ns (.88–2.32)	1.63 ns (.98–2.7)	1.73* (1.02–2.95)	1.71 ns (.99–2.96)
2.higher education					
Person-centred care (PACIC)		1.70*** (1.27–2.27)	1.55*** (1.14–2.11)	1.18 ns (.82–1.69)	1.01 ns (.69–1.49)
Continuity of care:					
Family/regular doctor 1 = no (Ref.), 2 = yes			1.12 ns (.65–1.94)	0.98 ns (.55–1.73)	1.22 ns (.67–2.23)
Family/regular nurse 1 = no (Ref.), 2 = yes			2.01** (1.20–3.36)	1.98* (1.16–3.4)	1.85* (1.06–3.23)
Social support:					
Community				1.55* (1.04–2.3)	1.59* (1.06–2.39)
Family and friends				1.43* (1.05–1.93)	1.20 ns (.87–1.66)
Peers				1.17 ns (.95–1.44)	1.15 ns (.92–1.43)
Diabetes-related distress					.50** (.31–.81)
Energy/vitality					1.02* (1.00–1.03)
Nagelkerke RSquare					.29 280
n					

*OR* odds ratio; *CI* confidence interval; *Ref*. reference group

*** *p* ≤ .001; ** *p* < .01; * *p* < .05; ns. non-significant

In the middle age group (55–64 years, Table 5), PACIC was positively associated with empowerment when controlling for all included variables. In the final model, a higher professional education, PACIC, all diabetes-related social support variables, and higher energy levels were positively and diabetes-related distress negatively associated with empowerment.

**Table 5:** Multivariate logistic regression models on determinants of empowerment among patients with T2DM, age group: 55–64 years

	Model 1 OR (95% CI)	Model 2 OR (95% CI)	Model 3 OR (95% CI)	Model 4 OR (95% CI)	Model 5 OR (95% CI)
Sex:					
1. men (Ref.)	1.08 ns. (.81–1.45)	1.14 ns. (.85–1.54)	1.11 ns (.82–1.5)	1.13 ns (.82–1.55)	1.29 ns (.92–1.80)
2. women					
Marital status					
1.single/widowed/divorced (Ref.)	1.29 ns. (.95–1.76)	1.22 ns. (.89–1.68)	1.23 ns (.89–1.69)	0.88 ns (.61–1.26)	0.79 ns (.54–1.15)
2. married/cohabiting					
Professional education					
1. lower education (Ref.)	1.36* (1.02–1.82)	1.40* (1.04–1.89)	1.48* (1.09–2.01)	1.74*** (1.25–2.41)	1.80*** (1.28–2.52)
2. higher education					
Person-centred care (PACIC)		1.79*** (1.49–2.16)	1.70*** (1.41–2.06)	1.26* (1.02–1.56)	1.32* (1.05–1.65)
Continuity of care:					
Family/regular doctor 1 = no (Ref.), 2 = yes			1.23 ns (.88–1.73)	.93 ns (.65–1.34)	.84 ns (.58–1.23)
Family/regular nurse 1 = no (Ref.), 2 = yes			1.26 ns (.92–1.71)	1.19 ns (.86–1.65)	1.18 ns (.84–1.66)
Social support:					
Community				1.80*** (1.40–2.30)	1.59*** (1.22–2.06)
Family and friends				1.46*** (1.23–1.75)	1.32** (1.10–1.59)
Peers				1.19* (1.04–1.36)	1.18* (1.03–1.36)
Diabetes-related distress					.43*** (.31–.59)
Energy/vitality					1.01** (1.01–1.02)
Nagelkerke RSquare n					.28 780

*OR* odds ratio; *CI* confidence interval; *Ref*. reference group

*** *p* ≤ .001; ** *p* < .01; * *p* < .05; ns. non-significant

In the oldest age group (65–75 years, Table 6), as well, PACIC was independently associated with empowerment when controlling for all included variables. In the final model, female sex, PACIC, community support and possibilities to influence, family and friend support and higher levels of energy were positively and diabetes-related distress negatively associated with empowerment.

**Table 6:** Multivariate logistic regression models on determinants of empowerment among patients with T2DM**,** age group: 65–75 years

	Model 1 OR (95% CI)	Model 2 OR (95% CI)	Model 3 OR (95% CI)	Model 4 OR (95% CI)	Model 5 OR (95% CI)
Sex:					
1. men (Ref.)	1.40* (1.06–1.85)	1.49** (1.12–2.0)	1.47** (1.10–1.97)	1.61** (1.19–2.18)	1.77*** (1.29–2.42)
2. women					
Marital status					
1. single/widowed/divorced (Ref.)	1.03 ns. (.76–1.39)	0.96 ns. (.70–1.31)	0.94 ns (.69–1.29)	0.78 ns (.56–1.09)	0.75 ns (.54–1.06)
2. married/cohabiting					
Professional education					
1. lower education (Ref.)	1.24 ns. (.93–1.64)	1.29 ns (.96–1.72)	1.32 ns (.99–1.77)	1.34 ns (.99–1.81)	1.34 ns (.98–1.82)
2. higher education					
Person-centred care (PACIC)		1.89*** (1.56–2.29)	1.85*** (1.52–2.25)	1.45*** (1.16–1.80)	1.47*** (1.18–1.84)
Continuity of care:					
Family/regular doctor 1 = no (Ref.), 2 = yes			1.33 ns (.97–1.82)	1.23 ns (.88–1.71)	1.25 ns (.89–1.75)
Family/regular nurse 1 = no (Ref.), 2 = yes			0.95 ns (.71–1.27)	0.86 ns (.63–1.16)	0.92 ns (.68–1.26)
Social support:					
Community				1.69*** (1.33–2.14)	1.45** (1.13–1.86)
Family and friends				1.36*** (1.14–1.62)	1.24* (1.03–1.49)
Peers				1.02 ns (.89–1.16)	1.01 ns (.87–1.16)
Diabetes-related distress					.49*** (.36–.68)
Energy/vitality					1.02*** (1.01–1.02)
Nagelkerke RSquare n					.22 883

OR: odds ratio; CI: confidence interval; Ref.: reference group

*** *p* ≤ .001; ** *p* < .01; * *p* < .05; ns. non-significant

To confirm the age group-wise differences found in the regression analyses, we also tested the interaction terms between main independent variables (including having a family/regular nurse) and age group on diabetes empowerment in adjusted regression models (data not shown). The interaction terms between all diabetes-related social support variables and age group on diabetes empowerment were statistically significant (*p* ≤ 0.001) when controlling for background, and quality of care variables (i.e. PACIC and continuity of care). Social support was significantly more important for empowerment among the two older age groups than the youngest, with the middle age group having higher odds ratios than the oldest. The interaction term between having a family/regular nurse and age group on empowerment was significant (*p* < 0.05) when controlling for background variables: it was significantly more important for the youngest age group as compared with the oldest. The interaction term between PACIC and age group on empowerment was not statistically significant.

## Discussion

In a large register-based sample of adults with T2D, we found age differentials in empowerment and in all variables proposed as possible correlates of empowerment. A significantly higher proportion of the youngest age group (27–54 years) had a low sense of empowerment, that is, a low sense of diabetes-related psychosocial self-efficacy. The youngest age group also reported significantly lower emotional wellbeing and more psychosocial distress than the two older age groups, and significantly less diabetes-related social support than the oldest group. With regard to quality of chronic illness care, the youngest age group reported less continuity of care, they reported less continuity of care, but a higher level of person-centred care. Furthermore, our findings are, to our knowledge, the first to show that community support and possibilities to influence was independently and consistently associated with higher diabetes empowerment across age groups, as was person-centred care in the two older age groups.

Loss of one’s sense of internalized security, like control over one’s body and emotions, as well as loss of one’s social and personal identities are seen as main factors behind patients’ feelings of powerlessness [43]. Feelings of powerlessness might also rise when becoming aware of the life changes needed [44]. In our study, the higher proportion of low empowerment scores among the youngest age group might mirror feelings of powerlessness. In this age group, a majority (70%) had early-onset diabetes (when defined as onset of diabetes ≤45 years) – which has been associated with a more aggressive disease with an increased risk of complications [4]. In accordance with this, the youngest age group had the lowest proportion of oral diabetes medication only – implying that their diabetes was harder to control. Moreover, the youngest age group had higher life stress and higher diabetes-related distress levels, and both lower vitality (energy) and emotional well-being than the two older age groups. These findings are in line with previous studies that found a higher prevalence of emotional problems [5] and an increased risk of psychosocial distress and depression among younger adult patients with T2D [6, 7, 45] – at the same time that they have a harder time managing their diabetes [7, 45]. The higher risk of emotional problems and psychosocial distress among younger adults could be due both to disease course and life-context related factors, like demands of working life and parenting, as suggested also by Hessler et al. [7].

Thus, it seems that younger T2D patients may require more psychosocial and self-management support than their older counterparts [45]. Studies have found that CCM-based interventions in primary care have been effective in improving both clinical, behavioural and psychosocial outcomes in patients with diabetes [27, 46]. In our study, the overall mean scores for PACIC were rather low, indicating a modest level of person-centred coordinated care. However, the ratings were higher among the youngest age group, suggesting that primary healthcare in Finland, at least to some extent, acknowledges the higher need for psychosocial and self-management support among the younger adult patients with T2D. Still, in our analyses, PACIC scores were not associated with empowerment in the youngest age group when controlling for the social support variables. In this age group, PACIC and community support and possibilities to influence correlated quite strongly (0.55; *p* < 0.001), and more strongly than in the other age groups (when in additional analysis checking correlations according to age groups), which might have affected the findings. Also, the interaction term between PACIC and age group on empowerment was not statistically significant, implying that there is no difference in this regard between the age groups. This said, we also checked the interaction between PACIC and age as a continuous variable on empowerment, and this interaction was statistically significant, showing that with increasing age the effect of PACIC on empowerment was stronger. This might suggest that healthcare needs as relates to person-centred coordinated care among the younger adults with T2D are different from the older ones. More studies are needed to confirm this.

Moreover, earlier studies show that healthcare professionals’ self-management support seem to focus more on medical and behavioural management than on helping patients deal with the emotional consequences of having a chronic disease [47]. The youngest age group in our study, besides having lower psychosocial wellbeing, perceived having less social support from all support sources, that is, family and friends, peers and the community. This, as well, points to a growing need in primary healthcare, and in the community, for paying attention to life context, and to different stages of adult life and how that might be related to disease status and diabetes management [7]. Our findings, for example, showed that having a family/regular nurse was independently associated with empowerment among the youngest age group. Earlier studies have found that patients with chronic diseases who also had a nurse involved in their care perceived their quality of care to be better, though only PACIC scores, and not nurse involvement, was independently associated with better self-management [48]. In our study, as well, PACIC, but neither having a family doctor nor nurse, was independently associated with empowerment in the two older age groups, whereas, in the youngest age group, both PACIC and having a family nurse were associated with empowerment when not yet including the social support variables. One explanation to this might be that the care provided by nurses might have been different for the youngest age group as compared to the older age groups in a way that is not reflected in the PACIC measure. Moreover, current health promotion programs and groups might be tailored more for older patients and their needs, and thus visits to a family/regular nurse might have been more important for the youngest age group.

The present findings are in line with earlier studies [21] showing that perceived social support and CCM-concordant care, i.e. person-centred coordinated care, seem to be independently and positively associated with sense of empowerment. Our study adds to these findings by showing the importance of community support, which was in all age groups independently associated with higher empowerment. Community resources are an important part of the CCM, where partnerships between primary healthcare and communities has been emphasized [14], but not much studied. However, a study by King et al. [11], in one healthcare organization in the USA, found that community support was independently associated with both diet and physical self-management among overweight adults with T2D. We also included possibilities to influence health and quality of life issues in the community, which is a central component of empowerment according to WHO [24, 25]. We found that community support and possibilities to influence was as important for the outcome, that is, for sense of empowerment, as person-centred care. In the youngest age group, community support and possibilities to influence, but not person-centred care, was independently associated with empowerment. However, as noted earlier, in this age group, the two former variables correlated mutually quite strongly, which might have affected the findings.

Additionally, in all age groups, diabetes-related distress was inversely and energy or vitality positively and independently associated with sense of empowerment, suggesting the need for psychosocial support and self-management support to handle emotional problems and stress. Earlier research suggests that interventions, which reduce diabetes-related distress and increase empowerment or self-efficacy might benefit not only psychological, but also metabolic outcomes among patients with T2D [6, 9].

In sum, patient empowerment and CCM-concordant care evaluated from the perspective of patients have both been proposed as quality indicators of chronic illness care [13, 49]. This is in accordance with the general emphasis in current health policy on, firstly, the patient perspective to quality of care, and secondly, a reorientation of health services from compliance towards increasing empowerment [12, 13]. Findings from the current study suggest that strengthening person-centred care (i.e. CCM-concordant care) and strengthening community resources – for example by increasing cooperation between primary healthcare and the community, as suggested by the CCM – in addition to strengthening the possibilities to influence health and quality of life issues in the community, may facilitate empowerment among adults with T2D. Moreover, the situation of younger adults with T2D and their probable higher need of psychosocial and self-management support as well as continuity of care in primary healthcare – especially as regards family/regular nurses – needs to be highlighted. Our analyses of possible interactions between age groups and correlates of empowerment confirmed that there seems to be age differences in correlates of empowerment, and we suggest that studies among adults with T2D need to consider age to get more insight into differences in healthcare needs.

### Strengths and limitations

The most important limitation of this study is its cross-sectional design, which implies that the observed associations can at least partially represent bi-directional influence. For instance, those with higher level of empowerment may perceive their quality of care as better. Moreover, the age-specific associations might also reflect the severity of T2D – as we discussed concerning the youngest age group – and not solely age effects. This study is based on data collected 9 years ago, when the development work to implement the CCM had quite recently started in selected healthcare centres in Finland. Thus, there might be improvements, which could be reflected in higher perceived person-centred care today, though the model has not yet made a clear impact on the Finnish care of diabetes. However, the characteristics we have identified as being associated with empowerment are likely to still apply. Empowerment is a gradual process and supporting psychological and social mechanism are not likely to change very quickly.

One important strength of the study is the representative sample and the reasonably high overall response rate. However, the lower response rate in the youngest age group calls for caution, and bias due to non-response is possible. Still, we have found that our data were highly reliable when compared with national register data on, for example, duration of diabetes, age of diagnosis, BMI and HbA1c-values [50]. Moreover, the large sample size made it possible to explore differences according to age groups. Self-reports represent a cost-effective data collection method but the responses can be biased to the socially desirable direction with regard to social support, diabetes related stress and energy or vitality.

Another strength to mention is that we were able to adjust for a wide variety of important potentially confounding sociodemographic and life-contextual factors. Hence, despite the cross-sectional study design the significant associations found reflect at least partially inherent and independent associations.

## Conclusions

This study, among adult patients with T2D, and with a special focus on patient age, found that community support and possibilities to influence community health issues was consistently and independently associated with higher empowerment in all age groups, as was person-centred primary healthcare in the two older age groups, thus supporting assumptions of the CCM and empowerment literature. Moreover, our findings highlight age-related differences, which suggest that primary healthcare and communities should place a special focus on meeting the psychosocial and self-management support needs of younger adults with T2D.

## Supplementary Information

**Additional file 1.** English translations of questionnaire instruments developed by the authors.

## Data Availability

A license for collecting the data through SII was granted for the present study. The data that support the findings of the study are covered by the granted permission, and so are not publicly available. However, permission can be requested from the SII. After a granted permission, request for the data can be sent to the authors.

## Abbreviations

CCM: Chronic Care Model
PACIC: Patient Assessment of Chronic Illness Care
T2D: Type 2 Diabetes
