# Supplementary material for: Empowerment among adult patients with type 2 diabetes: age differentials in relation to person-centred primary care, community resources, social support and other life-contextual circumstances
Source: BMC Public Health. 2021 May 1;21:844. doi: 10.1186/s12889-021-10855-0 (PMC8088546; doi:10.1186/s12889-021-10855-0)
Supplement: Supplementary file 1 — Additional file 1. English translations of questionnaire instruments developed by the authors. [file 12889_2021_10855_MOESM1_ESM.doc]

Additional file 1.

Empowerment among adult patients with type 2 diabetes: age differentials in relation to person-centred primary care, community resources, social support and other life-contextual circumstances

Nina Simonsen, Anne M Koponen, Sakari Suominen

Supplementary Material: English translations of questionnaire instruments that were developed by the authors for this study. The original questions were in Finnish. The translations to English are unofficial, i.e. not translated and back translated.

Author Note

Correspondence concerning this article should be addressed to Nina Simonsen, Folkhälsan Research Center, P.O.Box 211, 00251 Helsinki, Finland. E-mail: nina.simonsen-rehn@helsinki.fi

Diabetes counselling:

Have you, in your principal primary healthcare centre, gotten information, advice and guidance related to….. (Please choose one alternative on each line)

Answered on a 3-point scale: 1= not at all; 2= not sufficiently; 3= sufficiently, and the possibility to answer 4= does not concern me

1. Diabetes as a disease
2. Diabetes medication use
3. Use of other medication than diabetes medication
4. Use of vitamins and micro-nutrients
5. Healthy food, or food suitable for you
6. Quality of oils and fats and their use
7. Use of salt
8. Use of sugar
9. Physical activity suitable for you
10. Smoking and its health effects
11. The harm of alcohol and other addictive substances
12. Foot care
13. Rehabilitation services suitable for you
14. Weight control
15. Mental wellbeing

Diabetes-related social support:

The 12-item Social support scale of Toljamo [37] was used. We added three items to the scale, which are shown here.

Answered on a 5-point scale: ‘strongly disagree’ to ‘strongly agree’

1. In my municipality, people with diabetes get enough overall support
2. In my community, there are enough programs or activities to help with my diabetes
3. I have good possibilities to influence factors related to health and life quality in my community

Diabetes-related distress:

How often do the following activities feel burdensome or difficult? (Please choose one alternative on each line)

Answered on a 4-point scale: 1= almost never; 2= sometimes; 3= often; 4= almost always, and the possibility to answer 5= does not concern me

1. Monitoring your blood-sugar levels
2. Measuring your blood pressure
3. Taking your medicine
4. Checking your feet
5. Eating healthily
6. Engaging in physical activity

Life stress:

Please assess how much stress you have perceived during the last year (12 months) in the following life areas. (Please choose one alternative on each line)

Answered on a 4-point scale: 1= none; 2= some; 3= much; 4= very much, and the possibility to answer 5= does not concern me

1. Your own health
2. The health of your family members or friends
3. Work or unemployment
4. Living arrangements (e.g. moving, crowdedness)
5. Hurry (e.g. too much work, too little rest)
6. Your economic situation
7. Your intimate relationship
8. Your relationship with your children
9. Your relationship with other family members or friends
10. Death of a close person
